# Heat-Dependent Desorption of Proanthocyanidins from Grape-Derived Cell Wall Material under Variable Ethanol Concentrations in Model Wine Systems

**DOI:** 10.3390/molecules24193561

**Published:** 2019-10-01

**Authors:** Jordan W. Beaver, Konrad V. Miller, Cristina Medina-Plaza, Nicolas Dokoozlian, Ravi Ponangi, Thomas Blair, David Block, Anita Oberholster

**Affiliations:** 1 Department of Chemistry and Biochemistry, University of Texas at Tyler, 3900 University Blvd, Tyler, TX 75707, USA; jbeaver@uttyler.edu; 2 Department of Viticulture and Enology, University of California, One Shields Avenue, Davis, CA 95616, USA; vonmiller@ucdavis.edu (K.V.M.); cmedinaplaza@ucdavis.edu (C.M.-P.); deblock@ucdavis.edu (D.B.); 3 E&J Gallo Winery, 600 Yosemite Blvd, Modesto, CA 95354, USA; nick.dokoozlian@ejgallo.com (N.D.); ravi.ponangi@ejgallo.com (R.P.); tom.blair@ejgallo.com (T.B.)

**Keywords:** proanthocyanidins, desorption, grape, wine, fermentation, temperature, ethanol, cell wall material, modeling

## Abstract

Desorption of proanthocyanidins (PA) from grape cell wall material (CWM) was investigated in solutions of varying ethanol concentrations and increasing temperature. The results reveal the reversibility of PA-CWM interactions and the role that temperature and ethanol concentration play in the extent of PA desorption. Sequentially raising temperature from 15 to 35 °C resulted in desorption of up to 48% of the initial adsorbed PA. A comparison to a phenolic extraction model showed significant differences between the predicted and actual amount of PA that desorbed from the CWM. This suggests that the initial conditions of temperature and ethanol concentration must be considered when estimating PA extraction in red wine production. Under typical winemaking conditions, a significant amount of PA may be irreversibly adsorbed if exposed to CWM at low temperature (i.e., cold soak). A compositional analysis suggests the selective desorption of large molecular weight PA from CWM under all experimental conditions. Additionally, a preferential desorption of skin-derived PA over seed-derived PA was noted in the absence of ethanol.

## 1. Introduction

**Figure 1:**
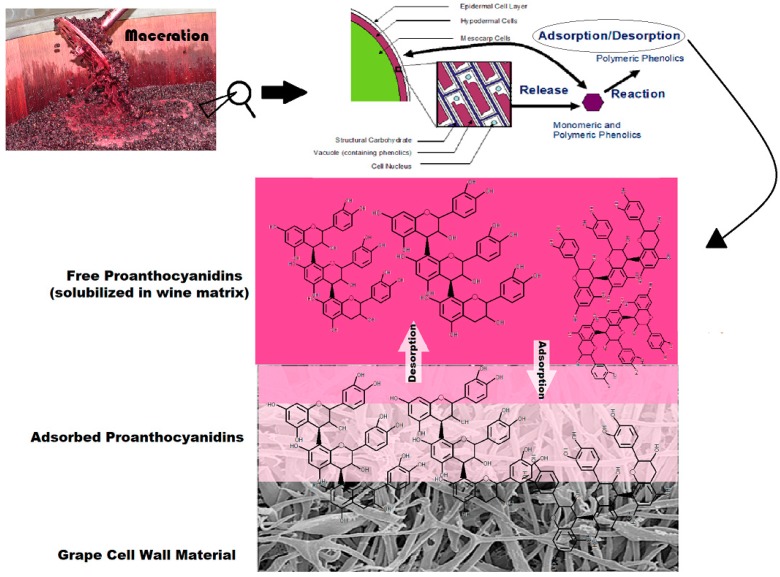
Representation of possible proanthocyanidins (PA) fate in red wine fermentation.

It is well established that proanthocyanidins (PA) are an essential component to the production of red wines. In addition to their potential benefit on human endothelial cell function as well as protection against oxidative damage, and anti-diabetic, anti-cholesterol, and anti-platelet functions [1], PA contribute directly to the taste, mouthfeel, and formation of stable pigment compounds in a finished wine, making them an important component in the production of high-quality vintages [2,3]. In general, the high extraction efficiency of polyphenols from grape skins and seeds during the maceration process is desirable. However, competing physical phenomena and reactions often detract from PA solubilization into the must and wine matrices (Figure 1). The adsorption of PA to the cell walls of solid grape material is one noted phenomenon that has been shown to significantly decrease PA concentration in the finished product [4]. Additionally, it has been suggested that this phenomenon contributes to the difficultly of accurately predicting levels of PA in a finished wine based on the PA content of the utilized grape berries [5].

Previous studies have investigated various aspects of PA adsorption to polysaccharide matrices, including grape-derived cell wall material (CWM). A 2006 study by Cartalade et al. examined the binding mechanisms of grape seed PA to solid surfaces of commercial, polysaccharide membranes [6]. In model wine systems of 12% ethanol and 20–1000 mg/L of isolated grape seed PA, PA-CWM interactions were found to be driven by increasing polar forces, suggesting that one of the primary adsorption interactions between PA and CWM is hydrogen bonding. Additionally, the desorption of PA induced by dilution was also performed, revealing the preferential recovery of flavan-3-ol monomers and PA with relatively small degrees of polymerization (DP). Le Bourvellec et al. further classified the types of bonding that occur in these interactions using CWM derived from apple skin. Hydrogen bonding and hydrophobic interactions were suggested as the main contributors [7]. The results from Bindon et al. and Beaver et al. showed a preferential adsorption of larger molecular weight PA over smaller molecular weight PA to grape skin derived CWM [4,8]. Additionally, it has been suggested that isolated PA in a model wine matrix seem to follow Langmuir Model assumptions of single-layer adsorption [8]. Additionally, the formation of PA-CWM interactions has been shown to be negatively correlated with increases in ethanol concentration in the solution and temperature of the liquid-solid system [8].

Recently, Miller et al. proposed an overall mechanistic model for phenolic extraction from grapes during red wine fermentation by utilizing the data from both model wine studies as well as practical winemaking trials [5]. One of the major components in the derivation of this extraction model was the estimation of PA adsorption to solid material in the fermentor. The predictive model by Miller et al. showed a good fit compared to winemaking trials. However, one assumption that the Miller model incorporates is the reversibility of PA adsorption (i.e., desorption of PA from CWM complex) as the temperature and ethanol change over the course and condition of a red wine fermentation. To date, however, the reversibility of PA-CWM adsorption phenomenon has yet to be investigated in a model wine system. In fact, the only known study to have investigated desorption of PA back into solution from PA-CWM complexes is that of Cartalade et al. [6]. This study, however, utilized commercial polysaccharide membranes as opposed to any plant-derived cell wall material which likely impacts the nature of the PA adsorption. Additionally, this study performed desorption by sequential dilution of the adsorbent material. In traditional winemaking, the dilution of the must or wine to promote PA desorption from cap material would likely not occur as this would increase the possibility of contamination as well as decrease the ethanol concentration of the finished product. As it stands, a more logical approach for analyzing PA desorption from CWM in a model wine system is to investigate the reversibility of the PA-CWM complexes under different temperatures and ethanol concentrations. Considering that many winemakers often utilize the variation in maceration temperature, such as cold soaking at low temperatures (approximately 10–15 °C) prior to fermentation or thermal vinification at higher temperatures, the necessity for understanding if and how PA will desorb from the solid material as a function of temperature and ethanol concentration seems apparent. Additionally, the elucidation of this phenomenon may aid in or substantiate the accuracy of predictive phenolic extraction models such as that of Miller et al. [5]. The following study aims to elucidate PA desorption from grape skin-derived CWM under conditions of varying temperature and ethanol concentration that are relevant to red wine production.

## 2. Results

### 2.1. Quantitative Analysis of PA Desorption

**Figure 2:**
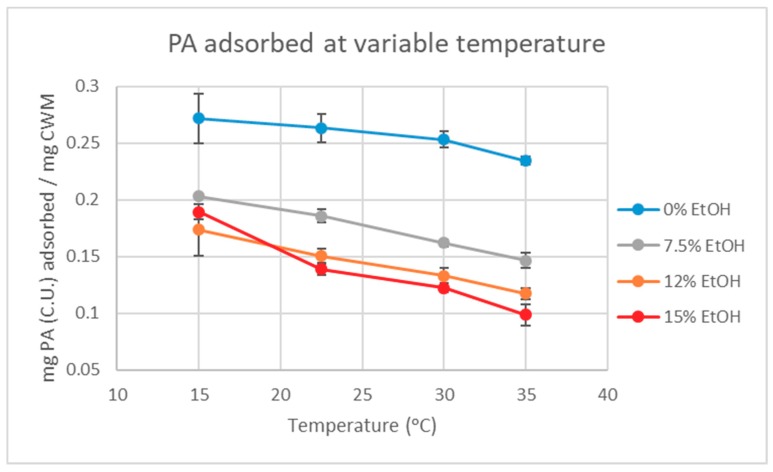
PA adsorption in four distinct model wine solutions over varying temperature conditions (*n* = 3 for each data point).

The PA solutions were initially exposed to CWM at 15 °C, then the temperature was sequentially increased. An aliquot of each trial and control sample was taken from the supernatant at 15, 22.5, 30, and 35 °C to calculate the concentration of PA in solution. The amount of PA lost to adsorption (in mg per mg CWM) was initially calculated by the difference in concentration between the control and trial samples. Figure 2 displays the amount of adsorbed PA under each ethanol condition (abbreviated “EtOH” in figures and tables) as the temperature increased in the system. The results showed that for all ethanol conditions there was a notable reduction in adsorbed PA over the increasing range of temperatures studied, supporting the hypothesis of temperature-dependent desorption of PA from CWM complexes.

Table 1 details the average percentages of PA desorption in each condition with respective standard deviations. The average desorption values were obtained by first calculating the amount of PA adsorbed at 15°C. This value was considered the maximum possible amount of recoverable PA. Then, as the temperature increased, the noted rise in PA concentration in solution was considered as a percentage of the bound PA desorbing from the CWM. For all of the sample sets, the average percentage of PA desorption increased with increases in the temperature and ethanol concentration. However, not all of the data was significant (denoted by capital and lower-case letters in Table 1). In particular, the variability of one sample within the 12% EtOH trials resulted in much of the standard deviation seen.

### 2.2. Comparison to Extraction Model

It has been previously suggested that PA adsorption to grape skin-derived CWM follows Langmuir model assumptions [5,8]. Equation (1) displays the Langmuir Equation as applicable to the studied system.

(1) $$q_{PA}\;=\;\frac{C_{PA}\times \;K_{eq}\times S_{CWM}}{1\;+C_{PA}\times \;K_{eq}}$$

q_PA_ = Mass of proanthocyanidin adsorbed per mass of CWM at equilibrium (mg PA (C.U.)/mg CWM)
C_PA_ = The concentration of proanthocyanidins in solution at equilibrium (mg PA (C.U.)/L)
K_eq_ = The equilibrium constant of the system (L/mg PA (C.U.))
S_CWM_ = The saturation point of the CWM (mg PA (C.U.)/mg CWM)

Miller et al. derived individual equations for estimating the equilibrium constant (K_eq_) and saturation point constant (S_CWM_) of the Langmuir Equation for PA adsorption as a function of temperature and ethanol. Equations (2) and (3) were derived using multi-parameter, curve fitting software [9]. The derivation of these equations was based on the data from adsorption tests in model wine that used PA concentrations between 500–1500 mg/L, incubation temperatures between 15–30 °C, and ethanol concentrations between 0–15% (*v*/*v*) [8].

(2) $$K_{eq}=\left(1.21\times 10^{-3}\right)+\frac{\left(-2.69\times 10^{-3}\times T\right)}{\left(-9.01\times T\right)+\left(-1.25\times 10^{2}\times E\right)+\left(T\times E\right)}$$

(3) $$S_{CWM}=\left(4.57\times 10^{-1}\right)+\frac{\left(2.04\times 10^{1}\times E\right)}{\left(-6.69\times E\right)+\left(4.57\times 10^{-1}\times T\right)+\left(T\times E\right)}$$

T = Temperature (Kelvin)
E = Percent Ethanol (% *v*/*v*)

Figures S1–S4 display the data points for each temperature and ethanol condition as a liquid-solid adsorption plot. The *x*-axis describes the concentration of PA in solution (mg PA per L), and the *y*-axis describes the concentration of PA adsorbed to the CWM (mg PA per mg CWM). Langmuir isotherms for each condition, calculated using Equations (2) and (3), are displayed on the respective plots. Figures S1–S3, which correspond to 0, 7.5, and 12% ethanol concentration systems respectively, show a good fit of the calculated model to the experimental data at the starting temperature of 15 °C. However, as the temperature increased in the systems, the data points fell further below the predicted extent of PA adsorption. In the 0% ethanol conditions, the average error between the data points and the predictive model at 35 °C was 0.05 mg PA adsorbed/mg CWM, which equates to an average percent error of approximately 18%. In the 7.5% ethanol conditions, the model overestimated adsorption by 0.08 mg PA adsorbed/mg CWM on average at 35 °C, an error of approximately 36%. The 12% ethanol condition was miscalculated by an average 0.05 mg PA adsorbed/mg CWM at 35 °C, equating to a 31% error between the data and the model. Ultimately, these results suggest that the proposed model underestimates the effect of temperature on the desorption of CWM-bound PA. Figure S4 displays the data and model for all 15% ethanol conditions. Unlike the other conditions, all the data points in these plots fell above the predicted level of adsorption. Additionally, the largest difference between the model at the observed results was within the conditions of initial exposure at 15 °C. The model underestimated adsorption by 0.07 mg PA adsorbed/mg CWM at this temperature which was an average percent error of 56%. At 35 °C, however, the model underestimated adsorption by only 0.03 mg PA adsorbed/mg CWM, an average error of 37%. Overall, this suggests that higher ethanol concentration does not play as significant a role in the desorption of CWM-bound PA as the current model proposes. Considering this noted underestimation of the temperature’s effect and overestimating ethanol’s effect on PA desorption, adjustments to the Miller et al. model could likely be made to further strengthen its predictive power.

### 2.3. Qualitative Analysis of PA Desorption

**Figure 3:**
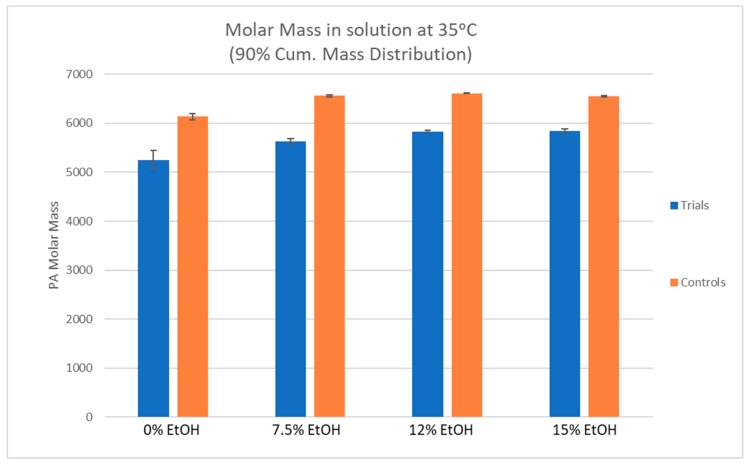
The results of gel permeation chromatography (GPC) analysis comparing molar mass (at 90% cumulative mass distribution) of trial and control solutions at the end of the desorption experiments (samples taken at 35 °C).

Figure 3 displays the molar mass in solution of each condition at the final incubation temperature of 35 °C. Molar masses were calculated from 90% cumulative mass distribution using gel permeation chromatography (GPC). Comparing the trial samples containing CWM with their respective control samples containing no CWM, there was a noted decrease in molar mass of the PA solutions under all conditions, suggesting a preferential adsorption of large molecular weight PA.

**Figure 4:**
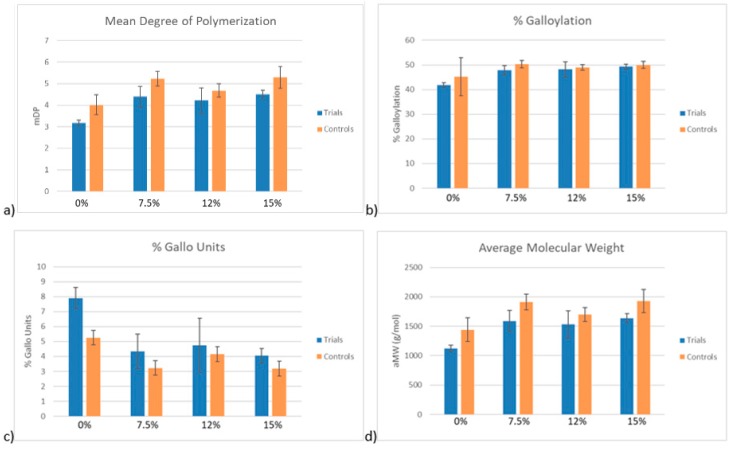
The results of phloroglucinolysis comparing PA composition in solution of trial and control samples at the end of the desorption experiment (samples taken at 35 °C). mDP—mean degree of polymerization; aMW—average molecular weight.

Figure 4 displays the data derived from phloroglucinolysis analysis of the PA solutions. As shown in Figure 4b, the % Galloylation was not significantly different between the control and trial samples. Figure 4c, however, showed an increase of approximately 3% in % gallo units comparing the trial to the control samples in the 0% ethanol condition. The average mean degree of polymerization (Figure 4a) and average molecular weight (Figure 4d) decreased in the trial samples in each condition compared to the average of the control samples. However, the data between the controls and trials was not significantly different in both 7.5% and 12% ethanol concentration conditions. It should be noted that phloroglucinolysis utilizes the ratio of free flavan-3-ol monomers to phloroglucinol-adduct monomers and thus can only be used to determine the average molecular weight and degree of polymerization of PA. Additionally, it is unable to cleave A-type PA. It was for these reasons that GPC was employed for additional determination of changes in PA size between the trial and control samples.

## 3. Discussion

The analysis of the quantitative data suggests that PA adsorption to CWM is, in fact, a reversible process driven by both changes in temperature and ethanol concentration, as indicated by Figure 2 and Table 1. However, even at the highest temperature and ethanol condition tested, the data showed that at least 52% of the initially adsorbed PA did not desorb from the CWM under finished wine conditions (15%EtOH-35 °C). By mass, this equates to approximately 0.95 mg (C.U.) of PA or approximately 15% of total available PA, remaining bound to the CWM by the end of the experiment. As the extent of PA adsorption to CWM is higher under low temperature and low ethanol concentration conditions, this has significant implications for winemakers that choose to utilize the cold soak method during red wine production. As hypothesized, both temperature and ethanol had a significant effect on the amount of PA that can be recovered from the CWM. However, under typical winemaking conditions, full recovery of adsorbed material is unlikely as increasing fermentor temperature past 35 °C is generally discouraged. Allowing temperature to reach above this point is known to increase the degradation of phenolic compounds as well as potentially produce undesirable aroma compounds from yeast [10,11,12,13]. To date, this is the first known, supporting evidence for temperature-dependent reversibility of the PA-CWM complexes in model wine systems. This supports the hypothesis of a system at equilibrium and further justifies the use of the Langmuir Model to predict PA adsorption in wine-like systems. This temperature and ethanol driven reversibility is due to an increase in PA solubility at higher temperatures and ethanol concentrations which acts as a competing interaction to PA-CWM complexation. This is likely driven by a higher thermodynamic stability of PA molecules in solution than complexing with the carbohydrate matrix of the adsorbent material. These findings are in agreement with theories made by previous studies [4,5,7,8], but would require a more in-depth characterization of PA-CWM bond complexes for full substantiation.

The PA adsorption component in the phenolic extraction model by Miller et al. showed a good fit to much of the experimental data under the conditions of initial exposure of the PA solution to the CWM, except within the case of the high ethanol condition. In the 0–12% ethanol conditions, the predictive model overestimated PA adsorption at higher temperatures, but the inverse was seen in the 15% ethanol condition. The error between the model and the experimental data was reduced at higher temperatures, making the prediction at 35 °C the most accurate compared to the 3 other temperature conditions within the 15% ethanol condition. Considering this apparent error, it would be advisable to take the impact of the initial temperature and ethanol conditions into consideration for the development or improvement of a winemaking phenolic extraction model.

**Figure 5:**
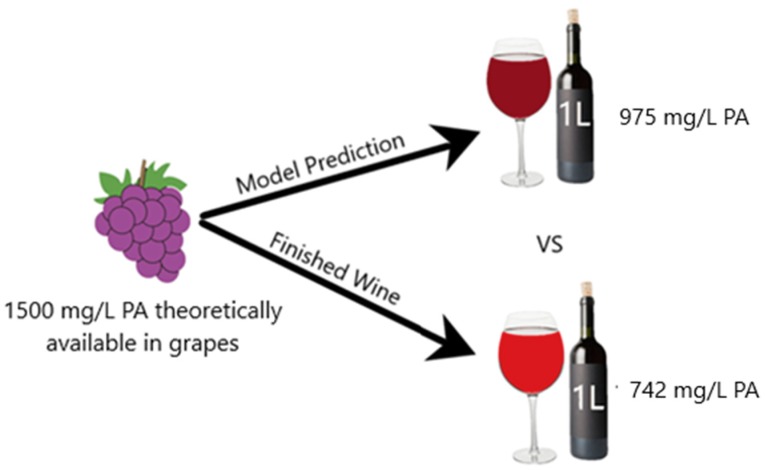
Theoretical representation of model prediction versus winemaking outcome disregarding impact from maceration.

For better comprehension of the difference between the predictive model and the real data, extrapolation of the results to winemaking conditions was performed (Figure 5). The production of 1 L of a standard red wine requires approximately 750 grape berries [14], and each berry contains approximately 10 mg of CWM [15]. If the PA concentration in each berry was approximately 1,500 mg/L, then the adsorption model would predict that approximately 525 mg of PA would be lost to CWM adsorption if fermentation reached a temperature of 35 °C and finished at an ethanol percentage of 15% (*v*/*v*). However, if cold soaking was performed at 15 °C prior to fermentation, the experimental data of this study suggests that the actual amount of PA lost to adsorption would be approximately 758 mg. Disregarding any other potential loss of PA, the actual PA concentration in the finished wine would be approximately 742 mg/L compared to the model prediction of 975 mg/L. This degree of difference would likely be sensorially perceivable in the taste, mouthfeel, and color of a red wine [16]. It should be noted, however, that this example disregards all other impacts of extraction from the model by Miller et al. [5]. In a realistic scenario, other factors of PA extraction—such as maceration time, cap temperature, cap management, and skin:seed ratio—would likely reduce the deviation between the model and the winemaking trial.

The percent reduction in PA molar mass between the control and trial PA solutions was similar between differing ethanol conditions, ranging from 10.8–14.5%. This suggests a preferential adsorption of larger molecular weight PA to CWM which is in agreement with several prior studies [4,8,10]. In previous work, PA solutions were exposed to grape skin CWM but the temperature of the system did not change [8]. The two most extreme conditions that were tested in that study were 0%EtOH-15 °C and 15%EtOH-30 °C. In the 0%EtOH-15 °C condition, the molar mass of the PA solution was reduced by approximately 40% after exposure to the CWM [7]. Under the condition of 15%EtOH-30 °C, the original PA solution saw an average decrease in molar mass of approximately 25% [9]. By comparison to the results of this study, the percent reduction in molar mass of every condition, regardless of ethanol concentration, was significantly lower. This suggests the desorption of high molecular weight PA from the CWM as temperature increases. Additionally, the increase in % gallo units between control and trial samples potentially suggests a preferential desorption of skin derived PA or preferential adsorption of seed derived PA under conditions of low ethanol and high temperature, though the change is minor. In the context of red wine production, these qualitative findings seem unlikely to impact sensorial perception of a finished product, though further study would be required to substantiate such a claim.

The formulas to estimate PA adsorption in Miller et al. were calculated from individual in-vitro adsorption experiments that varied temperature and ethanol concentration in separate trials as opposed to within the same samples [5]. The results from this study suggests that the initial conditions of PA exposure to CWM may impact the extent of desorption. A comparison of the experimental data to the proposed model showed that, for most conditions, the initial adsorption at 15 °C was accurately predicted. However, as temperature increased, the data points mostly fell further away from the predictive models. In the 0–12% ethanol conditions, the model tended to overestimate the extent of PA adsorption when the temperature of the system increased above 15 °C. Conversely, PA adsorption was underestimated in all 15% ethanol conditions by varying degrees. The Miller et al. model showed a good fit with practical winemaking data from Lerno et al. [11,12,13], but the results of the current study may suggest that, at higher ethanol concentrations (>12%), the calculated formula for PA adsorption may be estimating loss of PA from another reaction. Considering the year-to-year variability in the overall composition of red grapes, this formulaic inaccuracy may be exacerbated by currently unconsidered factors. Further study may be required to appropriately adjust the proposed model. However, it should be noted that the Miller model tends to overestimate adsorption of PA between ethanol concentrations of 0% to 12%. As previously stated, higher concentrations of polyphenolics are desirable in high-quality red wine, thus the overall difference between a predicted and finished red wine composition may ultimately be in the winemaker’s favor, if within these parameters. Additionally, high concentrations of PA were utilized for this study to ensure that the samples would fall within the limit of quantification of the analytical methods. However, not all varieties of grapes and wine contain such levels of PA, thus the deviations from the Miller model may be exacerbated by these high concentrations. Further study is needed to better understand the mechanistic aspects of PA interaction within wine fermentation systems to ensure the accurate modeling of red wine productions.

## 4. Materials and Methods

### 4.1. Reagents

Acetone (reagent grade), acetonitrile (HPLC grade), methanol (reagent grade), hydrochloric acid (37%, reagent grade), trifluoroacetic acid (TFA) (HPLC grade), formic acid (HPLC grade), sulfuric acid (96% reagent grade), phenol (reagent grade), l-ascorbic acid (molecular biology grade), HEPES buffer, phenol solution equilibrated with 10 mM Tris HCl buffer (pH = 7.5), potassium bitartrate (99%), (+)-catechin hydrate (98%), triethanolamine (99%), sodium dodecyl sulphate (99%), ferric chloride hexahydrate (99%), glacial acetic acid (99%), lithium chloride (99%), *N*,*N*-dimethylformamide (99%) and Toyopearl HW-50F size exclusion media were purchased from Sigma Aldrich (St. Louis, MO, USA). Koptec brand ethanol (95%) was purchased from Decon Laboratories, Inc. (King of Prussia, PA, USA). The deionized water was prepared in-house to a final purity of 18.2 MΩ. All the model wines used within the study contained 5 g/L potassium bitartrate and were adjusted to a pH of 3.5 using hydrochloric acid.

### 4.2. Instrumentation and Software

A Genesis 10S UV-Vis Spectrophotometer (Thermo Fischer Scientific, Waltham, MA, USA) was utilized for PA quantification. An Agilent 1260 Infinity HPLC (Agilent Technologies, Santa Clara, CA, USA) equipped with a diode array detector and a 6430 triple quadrupole system was used to analyze the phloroglucinolysis samples. An Agilent Poroshell 120 SB-C18 HPLC column (4.6 × 150 mm, 2.7 µm) was utilized for the phloroglucinolysis method. The same Agilent HPLC model equipped with the diode array detector was utilized for gel permeation chromatography (GPC) analysis. An Agilent OligioPore^®^ column (7.5 × 300 mm, 6 µm) and an Agilent MesoPore^®^ column (7.5 × 300 mm, 3 µm) were connected in series for the GPC analysis. The instrument control of the HPLC was performed using MassHunter^®^ software. Phloroglucinolysis analysis was performed using Agilent CDS ChemStation^®^, and WinGPC^®^ software (Polymer Standards Service, Philadelphia, PA, USA) was utilized for GPC analysis.

### 4.3. Isolation of Grape Skin Cell Wall Material

Skin-derived CWM was isolated from Thompson Seedless grape skins due to their lower phenolic content as well as the availability year-round. In addition, the phenolic extraction model by Miller et al. utilized this same source for isolating CWM used in their adsorption experiments [5]. This was done in order to optimize comparability of the collected data in this study to the adsorption models generated in the Miller et al. study. CWM was prepared using a modified method from Vidal, et al. [17]. The skins were manually separated from the flesh and ground under liquid nitrogen using an analytical mill (A11 Basic, IKA Works, Inc., Wilmington, NC, USA). The resulting material was extracted with 70% aqueous acetone (*v*/*v*) overnight at 4 °C to remove polyphenolics. This mixture was subsequently vacuum filtered (Whatman™ 1001–125 Grade 1 Qualitative Filter Paper, Diameter: 12.5 cm, Pore Size: 11 µm) to separate the solution from the solid material. The solids were washed with 70% aqueous acetone (*v*/*v*) until clear, followed by a deionized water wash. The solids were then extracted with 40 mM HEPES buffer (pH = 7) for one hour at room temperature in order to remove soluble polysaccharides. The mixture was again vacuum-filtered and washed with deionized water then pure acetone to remove residual HEPES buffer. The obtained solids were extracted with buffered phenol for 30 min at room temperature to remove cytoplasmic proteins. The mixture was vacuum filtered, washed with water, and subsequently washed with 80% aqueous ethanol (*v*/*v*) to remove residual phenol buffer. The resulting residue was then extracted with 50% chloroform in methanol (*v*/*v*) for 30 min at room temperature to remove lipids. The mixture was vacuum filtered again and washed with water and pure acetone to remove residual chloroform and methanol. The resulting solid material was pushed through a 500 µm mesh and finally stored under nitrogen at −20 °C until utilized.

### 4.4. Isolation of Proanthocyanidins

PA were isolated from the skins of Chardonnay grapes from Lodi, CA and from the seeds of Cabernet Sauvignon grapes from Napa, CA. Chardonnay skins were used in lieu of Cabernet Sauvignon skins as they were readily accessible and contained no anthocyanins. Additionally, these skins and seeds were the same source material utilized in the adsorption experiments used in the construction of Miller et al. model [5]. This was done in order to optimize comparability of the collected data in this study to the adsorption models generated by Miller et al. Chardonnay skins and Cabernet Sauvignon seeds were manually separated from the rest of the berry. PA were extracted separately using 70% aqueous acetone with 0.1% TFA (*v*/*v*). These mixtures were homogenized using a T18 digital ULTRA-TURRAX^®^ blender (IKA^®^ Works, Inc., Wilmington, NC, USA) and extracted overnight at 4 °C. The mixtures were then centrifuged at 3,220× *g* for 15 min, and the supernatant was drawn off. These solutions were concentrated under reduced pressure by rotoevaporation at 34 °C. The resulting aqueous solutions were then loaded onto separate low-pressure chromatography (LPC) columns of Toyopearl HW-50F (500 mL bed volume). The LPC method used for the isolation of proanthocyanidins is described by Kennedy et al. [18]. The eluates from the LPC columns containing the isolated PA were concentrated under reduced pressure, lyophilized, and stored at −80 °C until utilized.

### 4.5. In Vitro Desorption Experiment

The temperature and ethanol ranges utilized in these experiments were chosen for their applicability to winemaking conditions as well as for an appropriate comparison to the adsorption models presented by Miller et al. [5]. The temperatures below 15 °C are favorable when utilizing the technique of cold soaking, and temperatures above 35 °C are rarely seen as the yeast would generate undesirable amounts of esters [10,11,12,13], and phenolic compounds, such as anthocyanins, would degrade much more rapidly [19,20]. In general, the majority of wine fermentations are kept at approximately 25–30 °C, but a recent reactor engineering study showed that cap temperatures easily reach 35 °C between pump-overs and/or punch-downs [21]. Additionally, the majority of full-bodied red wine (such as Cabernet Sauvignon) are rarely higher than 15% ABV [22].

The isolated PA from grape skins and seeds were manually weighed, solubilized to a concentration of 1,500 mg/L, and combined in a 1:1 ratio. The PA solutions were made using model wine (pH 3.5) of varying ethanol concentrations (0, 7.5, 12, and 15% *v*/*v*). The solutions were then allowed to reach 15 °C in an incubator-shaker. Simultaneously, 10 mg of CWM was manually weighed into 5 mL Eppendorf tubes and allowed to reach 15 °C in the same incubator. Each PA solution (4.5 mL) was then pipetted into the Eppendorf tubes containing CWM. For each PA solution of varying ethanol concentration, trials were conducted in triplicate. Additionally, triplicate control samples, which contained the same PA solution but no CWM, were monitored throughout the experiment. Previous studies showed that the PA adsorption to CWM at low temperatures can take between 480 and 720 min to reach equilibrium [8], thus the mixture was left to react for 720 min. Afterwards, a 125 µL aliquot was taken from the supernatant of each trial and control sample and stored at −80 °C for later quantification. The temperature of the incubator-shaker was then increased to 22.5 °C and held at this temperature for 120 min. Another 125 µL aliquot was taken from the supernatant of each sample and stored. The incubator was then increased to 30 °C, and 125 µL was taken from each sample after 120 min. Lastly, the system was heated to 35 °C, and a final 125 µL aliquot was taken from each sample after another 120 min. A 1 mL sample of each PA solution was taken and lyophilized for later analysis by phloroglucinolysis and gel permeation chromatography (GPC).

### 4.6. Quantitative Analysis of Proanthocyanidin Solution

A quantitative analysis of the PA samples was performed using the ferric chloride assay for total iron-reactive phenolics [23]. A calibration curve was constructed using (+)-catechin hydrate as standard, thus the units of the PA samples are specified as mg catechin units (C.U.). The frozen samples were thawed and vortexed to ensure resolubilization of any PA that may have precipitated during storage. A 75 µL aliquot of each sample was transferred to a 1 mL cuvette and mixed with 800 µL of resuspension buffer (50 g/L sodium dodecyl sulphate in 5% triethanolamine- 95% deionized water, pH = 9.4). An initial reading of this mixture at 510 nm was taken to account for background phenolics. A 125 µL aliquot of ferric chloride reagent (2.7 g/L ferric chloride, 800 µL/L hydrochloric acid (37%) in deionized water) was added to the cuvette and vortexed. The absorbance of the mixture was measured at 510 nm, and the concentration of the PA solution at each timepoint was determined against the (+)-catechin calibration curve. The amount of PA adsorbed to the CWM in each trial was determined by the observed difference between the concentration of the trial samples (containing CWM) and the average concentration of the control samples (containing no CWM). The average adsorption of the triplicate trials at each temperature and their respective standard deviations is displayed in Figure 2. The amount of PA adsorbed to the CWM at 15 °C in each trial was considered the maximum amount of PA that could desorb back into the solution. The percent desorption at each incubation temperature above 15 °C (Table 1) was calculated by the increase in PA in the supernatant of the solution compared to the amount of PA bound at 15 °C.

### 4.7. Qualitative Analysis of Proanthocyanidin Solution

The lyophilized samples were manually separated into two fractions. The first of these fractions—to be used for phloroglucinolysis—was combined with 125 µL of acidified methanol (0.1 M HCl) and vortexed to resolubilize the dried PA. Phloroglucinolysis was performed by reacting PA samples with 50 µL of phloroglucinol reagent at 50 °C for 20 min. The reaction was quenched with 0.5 mL of sodium acetate (40 mM). The samples were centrifuged, transferred to amber glass vials, and analyzed via HPLC-DAD-MS/MS using a previously defined method [24]. A calibration curve using (+)-catechin as a standard was constructed. The molar absorptivities of epicatechin, epicatechin gallate, epigallocatechin, and their phloroglucinol adducts were applied to the catechin calibration curve to construct individual calibrations for each compound. The mean degree of polymerization (mDP), % galloylation (relating to flavan-3-ols containing a gallate ester group), and % gallo units (relating to flavan-3-ols containing trihydroxylated B-rings) were calculated from this method in each sample.

The second fraction of the freeze-dried samples was used for GPC analysis. Each sample was dissolved in 0.5 mL of GPC mobile phase (0.15 M lithium chloride, 5% deionized water, 1% glacial acetic acid, 94% *N*,*N*-Dimethylformamide). The Agilent OligioPore^®^ and MesoPore^®^ columns were connected in a series and equilibrated in the GPC mobile phase for one hour at a flow rate of 0.65 mL/min prior to first injection. Twenty µL of each sample was injected, and the isocratic flow of the mobile phase was maintained at a rate of 0.65 mL/min for 40 min. WinGPC^®^ software was used to analyze the cumulative molar mass distribution of each sample. The broad standards for constructing a calibration curve were isolated using a previously developed method from Cabernet Sauvignon seed isolate and Chardonnay skin isolate [18].

### 4.8. Statistical Analysis

The significant difference between the data sets was determined from triplicate samples by means of analysis of variance using the Data Analysis package in Microsoft Excel^®^. A significance level of α = 0.05 was chosen for each test, thus the significant difference between the data sets was defined by *p* < 0.05.

## Figures and Tables

**Table 1:** Average percent of PA desorption (compared to initial adsorption at 15 °C) and respective standard deviations as incubation temperature increased in distinct model wine solutions (*n* = 3).

Temp. (°C)	0% EtOH	7.5% EtOH	12% EtOH	15% EtOH
**22.5**	2.82 ± 3.39 ^A, a^	8.60 ± 2.01% ^A, a^	17.48 ± 1.46% ^A, b^	26.73 ± 0.37% ^A, c^
**30**	7.49 ± 5.51% ^AB, a^	22.30 ± 3.58% ^B, b^	25.24 ± 9.94% ^A, bc^	37.36 ± 1.71% ^B, c^
**35**	13.35 ± 5.62% ^B, a^	27.88 ± 3.38% ^B, b^	31.59 ± 10.26% ^A, abc^	47.96 ± 3.37% ^C, c^

Capital letters = significance between the same EtOH condition at different temperatures (*p* < 0.05). Lower case letters = significance between different EtOH conditions at the same temperature (*p* < 0.05).

## Supplementary Materials

Figure S1. Adsorption data points from individual conditions and Langmuir isotherms constructed from calculated values of Equation 2 and 3 in model wine systems of 0% ethanol (*v*/*v*). Figure S2. Adsorption data points from individual conditions and Langmuir isotherms constructed from calculated values of Equation 2 and 3 in model wine systems of 7.5% ethanol (*v*/*v*). Figure S3. Adsorption data points from individual conditions and Langmuir isotherms constructed from calculated values of Equation 2 and 3 in model wine systems of 12% ethanol (*v*/*v*). Figure S4. Adsorption data points from individual conditions and Langmuir isotherms constructed from calculated values of Equation 2 and 3 in model wine systems of 15% ethanol (*v*/*v*).
