# Supplementary material for: Heat-Dependent Desorption of Proanthocyanidins from Grape-Derived Cell Wall Material under Variable Ethanol Concentrations in Model Wine Systems
Source: Molecules. 2019 Oct 1;24(19):3561. doi: 10.3390/molecules24193561 (PMC6804194; doi:10.3390/molecules24193561)
Supplement: Supplementary file 1 [file molecules-24-03561-s001.zip › molecules-589324-supplementary.finalproof.docx]

Communication

Heat-Dependent Desorption of Proanthocyanidins from Grape-Derived Cell Wall Material under Variable Ethanol Concentrations in Model Wine Systems

Supplementary Material


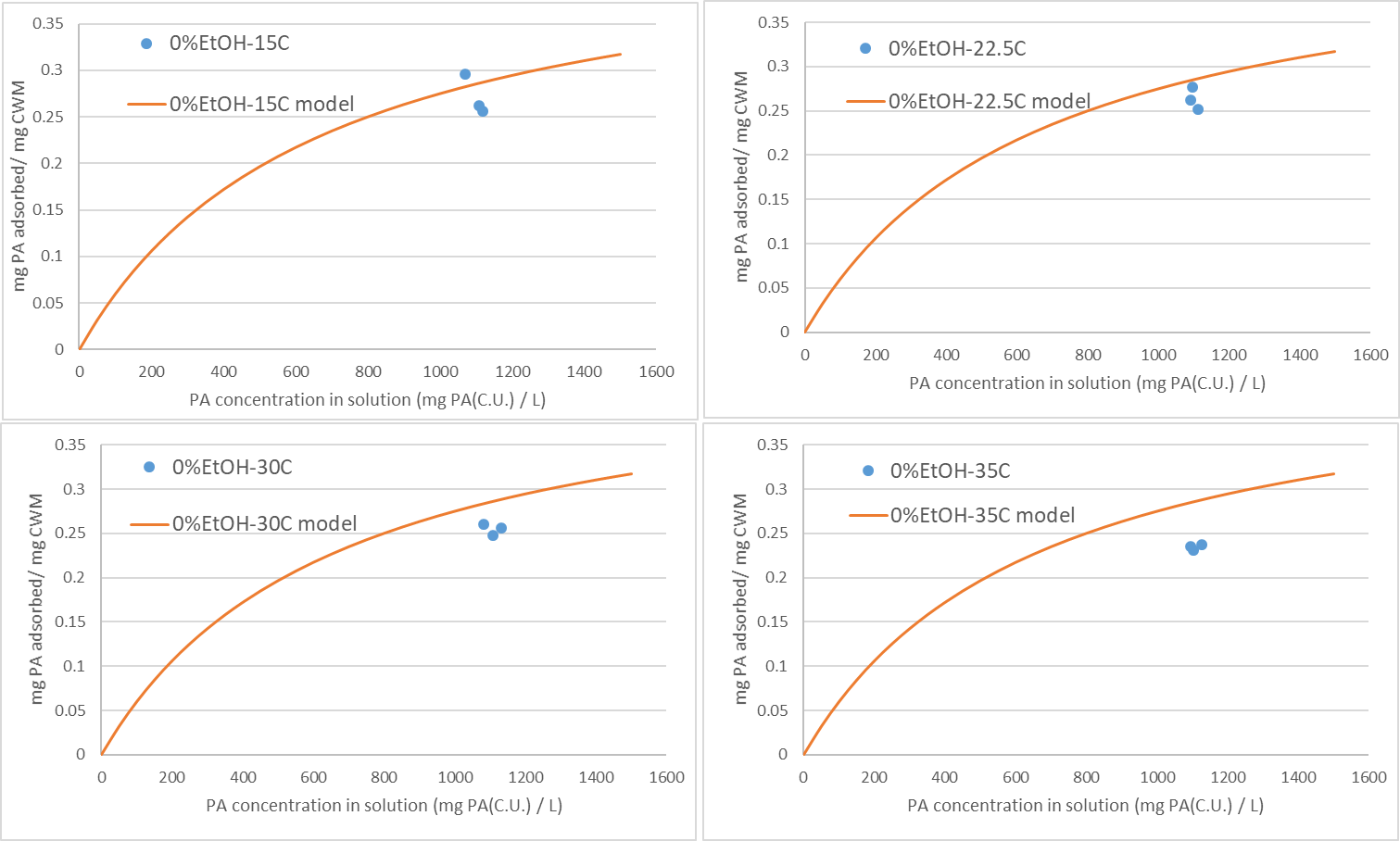


**Figure S1.** Adsorption data points from individual conditions and Langmuir isotherms constructed from calculated values of Equation 2 and 3 in model wine systems of 0% ethanol (*v*/*v*).


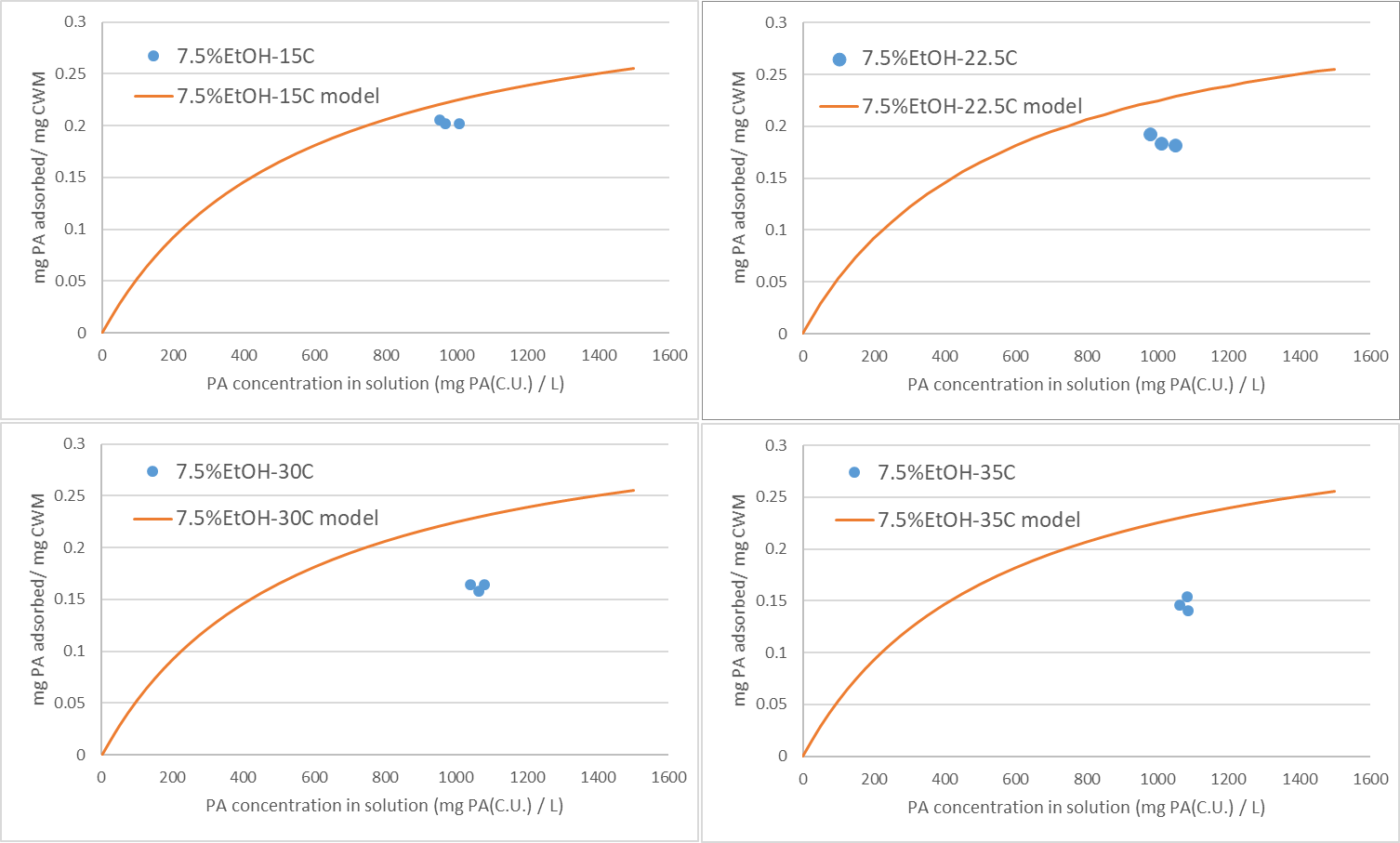


**Figure S2.** Adsorption data points from individual conditions and Langmuir isotherms constructed from calculated values of Equation 2 and 3 in model wine systems of 7.5% ethanol (*v*/*v*).


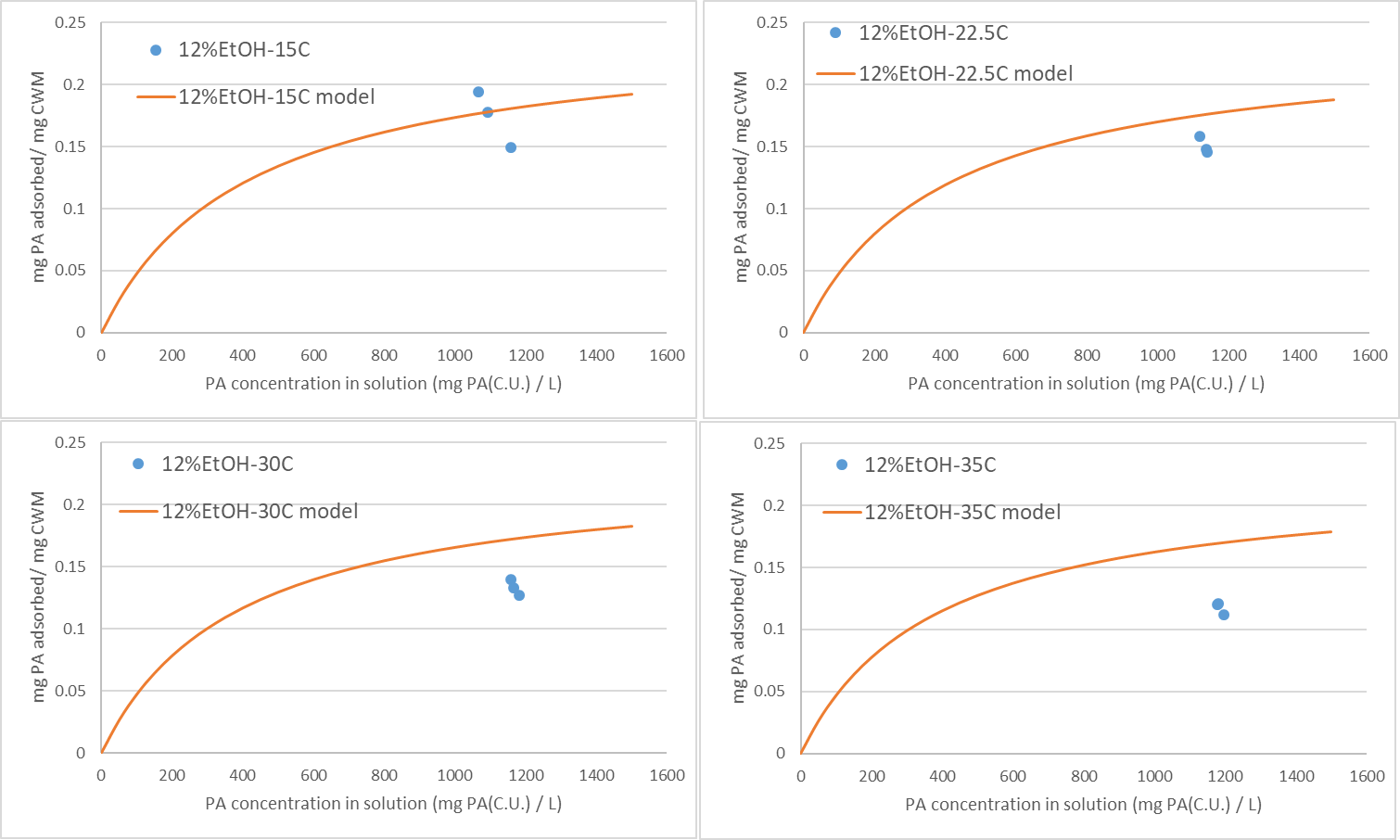


**Figure S3.** Adsorption data points from individual conditions and Langmuir isotherms constructed from calculated values of Equation 2 and 3 in model wine systems of 12% ethanol (*v*/*v*).


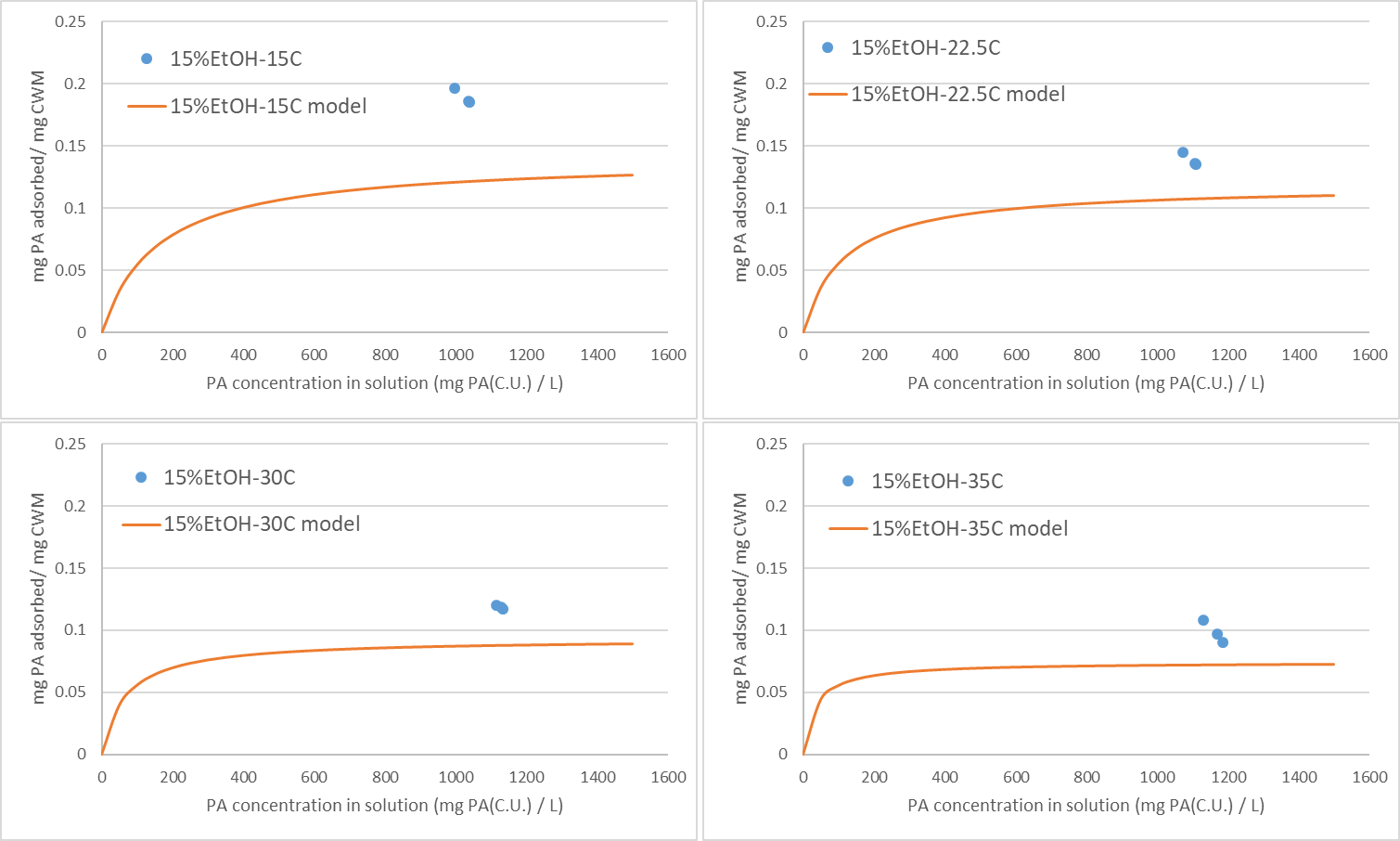


**Figure S4.** Adsorption data points from individual conditions and Langmuir isotherms constructed from calculated values of Equation 2 and 3 in model wine systems of 15% ethanol (*v*/*v*).
