# Supplementary material for: Heat-Dependent Desorption of Proanthocyanidins from Grape-Derived Cell Wall Material under Variable Ethanol Concentrations in Model Wine Systems
Source: Molecules. 2019 Oct 1;24(19):3561. doi: 10.3390/molecules24193561 (PMC6804194; doi:10.3390/molecules24193561)
Supplement: Supplementary file 1 [file molecules-24-03561-s001.zip › molecules-589324-supplementary.finalproof.pdf]

molecules

MDPI

Communication

Heat-Dependent Desorption of Proanthocyanidins from Grape-Derived Cell Wall Material under Variable Ethanol Concentrations in Model Wine Systems

Supplementary Material

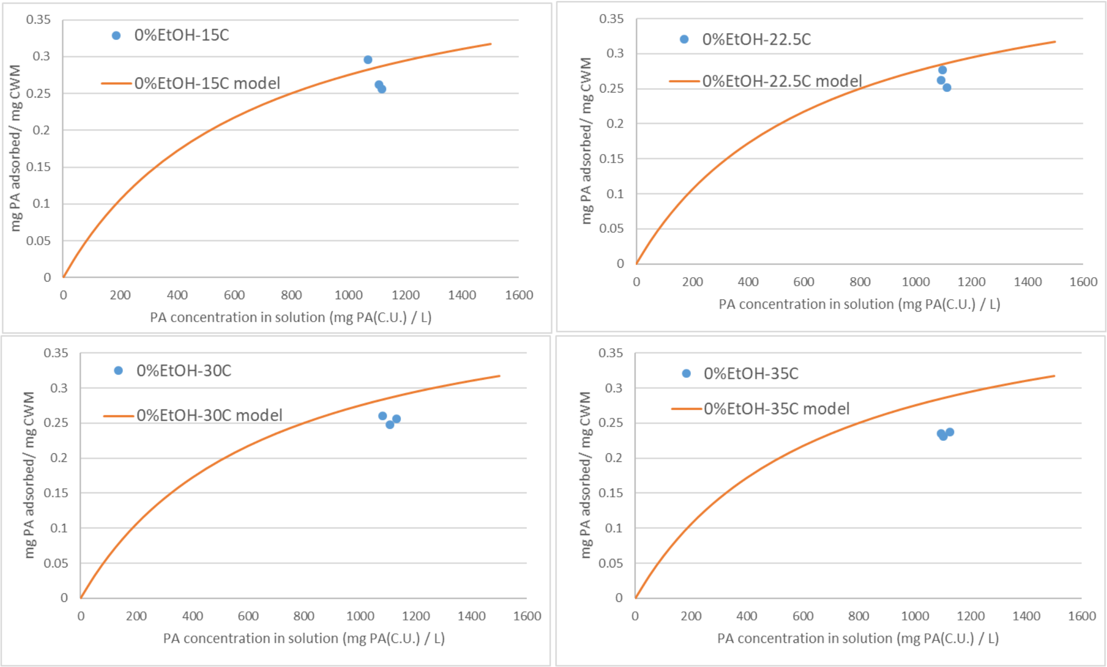

Figure S1. Adsorption data points from individual conditions and Langmuir isotherms constructed from calculated values of Equation 2 and 3 in model wine systems of 0% ethanol (*v*/*v*).

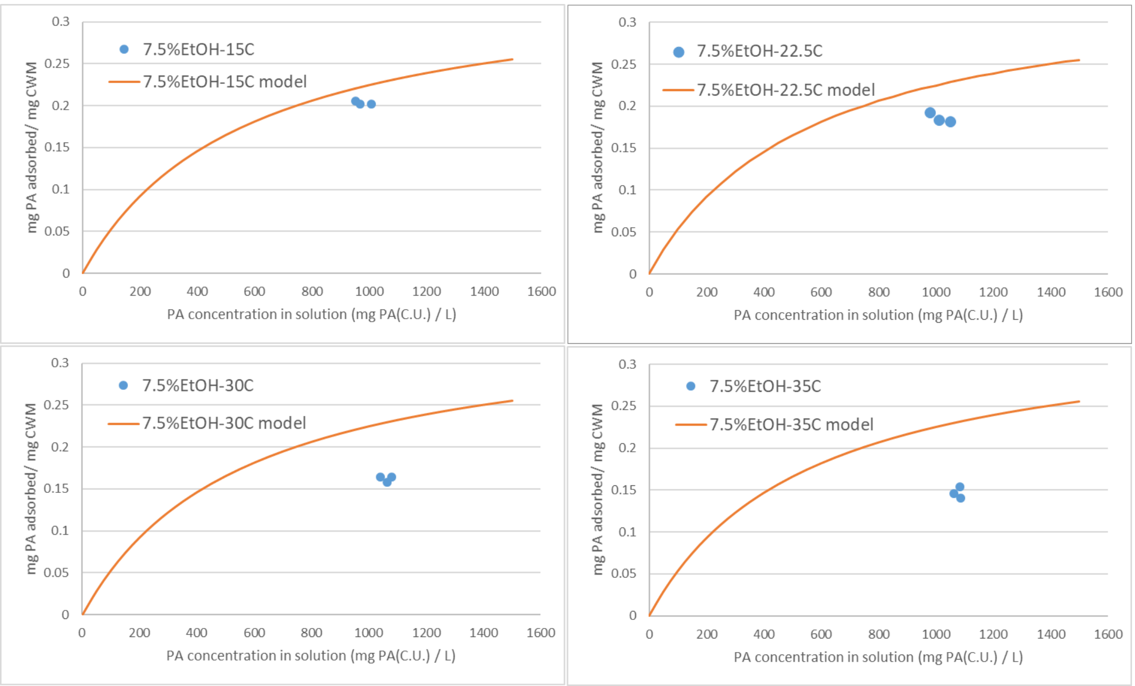

Figure S2. Adsorption data points from individual conditions and Langmuir isotherms constructed from calculated values of Equation 2 and 3 in model wine systems of 7.5% ethanol (*v*/*v*).

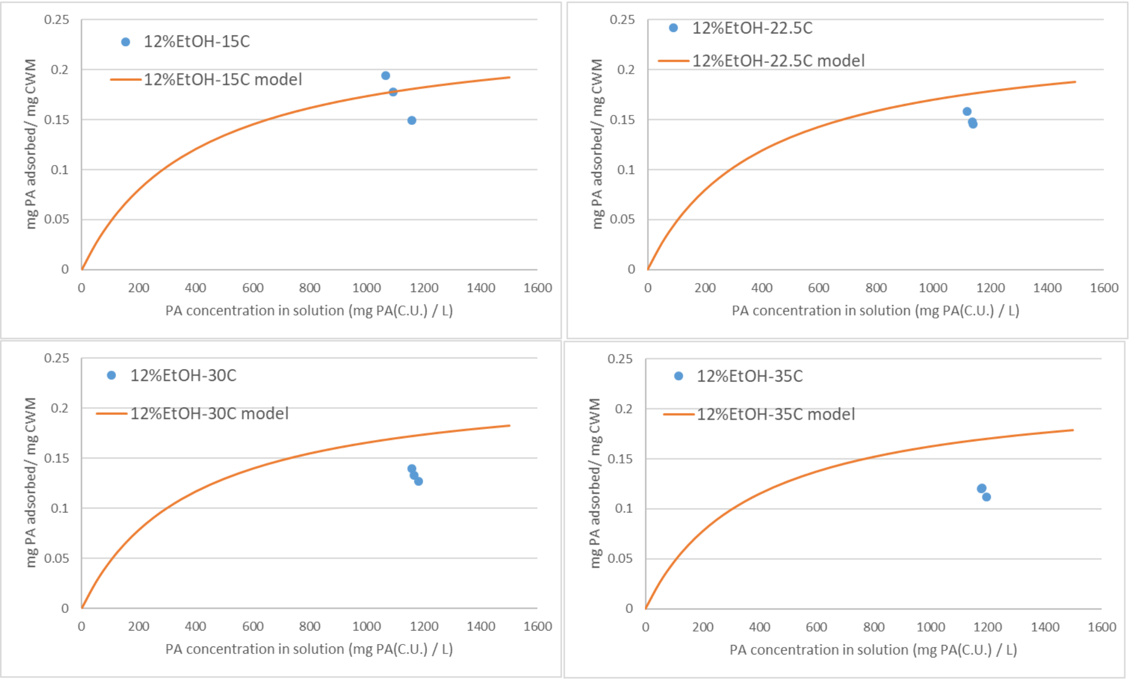

Figure S3. Adsorption data points from individual conditions and Langmuir isotherms constructed from calculated values of Equation 2 and 3 in model wine systems of 12% ethanol (*v*/*v*).

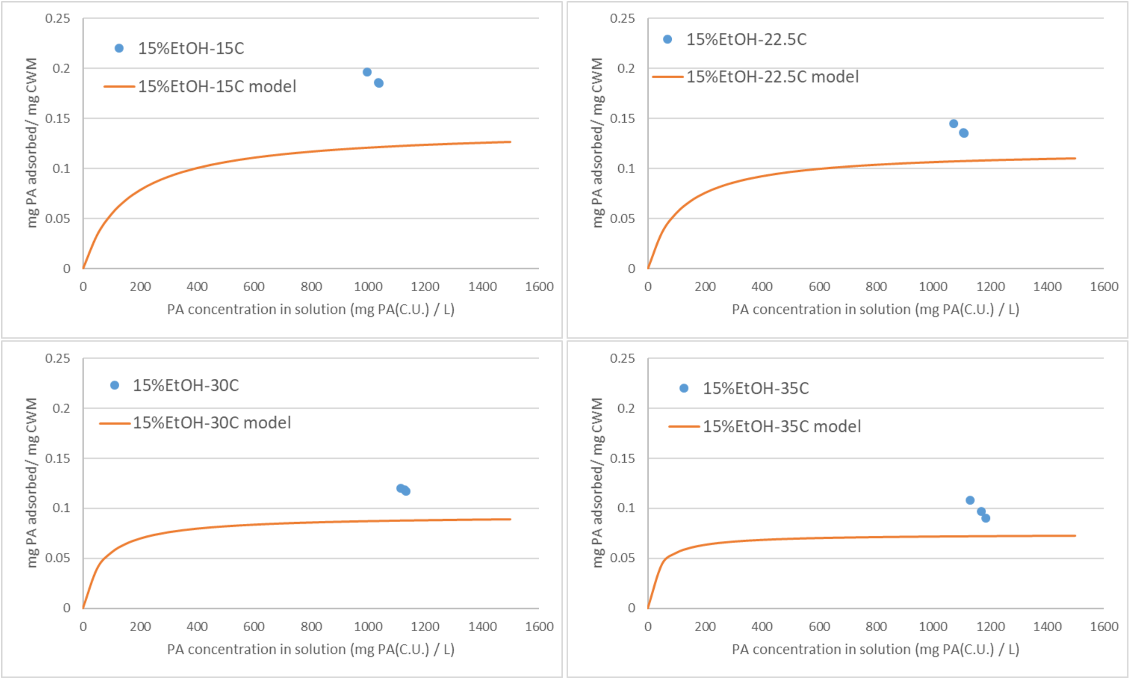

Figure S4. Adsorption data points from individual conditions and Langmuir isotherms constructed from calculated values of Equation 2 and 3 in model wine systems of 15% ethanol (*v*/*v*).
